# Mental Rotation: The Effects of Processing Strategy, Gender and Task Characteristics on Children's Accuracy, Reaction Time and Eye Movements’ Pattern

**DOI:** 10.16910/jemr.12.8.2

**Published:** 2019-11-06

**Authors:** Dorit Taragin, David Tzuriel, Eli Vakil

**Affiliations:** Bar-Ilan University, Hemdat Hadarom College, Israel; Bar-Ilan University, Israel

**Keywords:** Mental rotation, difficulty level, gender differences, global/local strategy, eye movement patternaugmented reality

## Abstract

The effects of gender, strategy and task characteristics on children's mental rotation (MR) behavioral measures and eye movements were studied. Eye movements reflect thinking pattern and assist understanding mental rotation performance. Eighty-three fourth-grade children (44 boys and 39 girls) were administered the Computerized Windows Mental Rotation test (CWMR) while having their eye movements monitored and completed a Strategy Self-Report (global/local/combined) and a Spatial Span (WM) subtest. Difficulty level affected performance and was reflected in a different eye movement pattern. Boys were more accurate than girls, but they did not differ in their eye movement pattern. Eye movement pattern was related to strategy, accu-racy and reaction time, revealing that the global and combined strategy were more effective compared with local strategy. WM was found to correlate with accuracy at the easy level of the test. The usage of eye movement measures assists in elaborating our knowledge regarding MR performance among chil-dren and enable a wider understanding regarding the interaction between gender, strategy and difficulty level.

## Introduction

### Definition and goals 

Mental rotation (MR) was defined as the ability to mentally transform two- or three-dimensional objects in space (1). It is a fundamental spatial thinking skill that is essential for academic as well as everyday tasks. The research literature is replete with findings indicating large individual and gender differences (e.g. 2, 3, 4).

The main purpose of the current study was to investigate the effects of gender, cognitive strategy (global/local) and task characteristics on children's eye movements and behavioral measures in MR tasks. In the following sections we review the relation of MR to cognitive strategies, characteristics of MR tasks, gender differences, working memory (WM) and eye movements. 

### MR and cognitive strategies 

MR [XMLmind] can be performed by usage of a global-holistic strategy, local-analytic strategy or a combined strategy (5, 6, 7). A global-holistic strategy refers to a gestalt-like process in which the entire shape is rotated as one unit. A local-analytic strategy refers to one-to-one correspondence between the target and the answers. A combined strategy involves both global and local strategies and is expressed in either rotating the entire shape with focus on its specific details or picturing several elements (but not all of them) at a time (6, 8).

The strategy used for MR tasks was found to correlate with accuracy and reaction time measures. There is evidence showing that the global-holistic strategy was associated with better MR performance, compared with the local-analytic strategy (5, 9). Yet, Nazareth and colleagues (7) found that the local strategy was more useful for MR tasks. 

In addition, for the *Mental Rotation Test* (MRT, 10), different characteristics of the target seemed to require different strategies; for most items, mainly items which include mirrored distracters, the global strategy led to more accurate performance compared with the local strategy. However, for several items in which the distracter figure could be excluded relatively easily by a feature comparison strategy, the local-analytic strategy was found to be effective as well (11).

### Characteristics of MR tasks

For MR tasks, different characteristics of the target and the task requirements were found to affect reaction time and accuracy. Reaction time increases linearly with angular disparity for same trials (1, 12), and depart from linearity such that the slops were shallower above 90 degrees (13). Raising the complexity level (e.g., number of components and foil type) decreased accuracy (13, 14).

An [XMLmind] for these differences may be related to the strategy participants used. Boone and Hegarty (13) found that among adults who performed the MRT, MR strategy was dominant for angles less than 90^0^, whereas more orientation independent strategies were used as the angular disparity increased. It seems that the increase in the rotation degrees increased the difficulty level and resulted in switching strategies. Similar findings were found by Khooshabeh et al. (5) when half of figures were manipulated to create "fragmented" figures. Good imagers used holistic strategy by default, and switched to a different strategy when needed, whereas poor imagers were less flexible and used a piecemeal strategy regardless to the task demands. 

Among children, the use of MRT led to a floor effect (2, 4), possibly because of the three-dimension complex items that were used. Therefore, in order to reveal individual differences among children, the MR tests that were often used were two-dimension tests (3, 14, 15), or three-dimension tests that have simple or familiar stimuli (16, 17).

In a two-dimension MR test, children with global spatial perception were more accurate compared with children who had local spatial perception (3). When three-dimension familiar female- or male-stereotyped figures were used (e.g., doll and hairbrush as female familiar figures and train and tractor as male familiar figures), faster reaction time was reported in gender-congruent conditions (e.g., boys with male familiar figures and girls with female familiar figures). In addition, the analytical strategies might have been as efficient as holistic strategies for gender-familiar objects, but the use of holistic strategy resulted in faster reaction time for incongruent gender-object conditions (18). It is possible that as the difficulty level increases (e.g., incongruent gender-object) it is more difficult for children to verbalize and memorize the target properties and therefore a global perception might be more helpful. 

Neuburger et al. (15) reported that performance in MR tasks with familiar and concrete stimuli seems to improve to a greater degree between 8 and 10 years than the MR performance in tasks with unfamiliar and abstract stimuli. The conclusion is that different characteristics of the task and target can result in different strategies and in different behavioral performance children (18) and adults (13).

In the current study, in order to observe children's' MR performance in two difficulty levels, and in order to avoid a floor effect and gender familiarity advantage, we used a two-dimensional task, with shapes (squares and triangles) that do not have any gender familiarity bias. The task is based on the "Windows Mental Rotation Test" from the Cognitive Modifiability Battery (3, 14, 19).

### Gender differences in MR tasks

Gender differences in MR tasks showed extensively over the years an advantage for men over women (e.g., 4, 20). Yet, there are controversial findings regarding the age in which these gender differences emerge. Some researchers (e.g., 21) showed that gender differences in MR exist already among infants, while others (e.g., 15) found that gender differences emerge only around the early adolescence years. An overview of gender differences in MR through childhood and adolescence years revealed that the magnitude of the gender differences increased with age (4, 22). Geiser et al. (22) sampled 1624 students, between the ages of 9 and 23, and found gender differences in MR tests in favor of males for all age groups. MR scores increased with age for both genders, but the increase with age was stronger for males than for females. Gender alone accounted for 16.1% of the variance in latent MR test scores (22). Neuburger et al. (15) who compared the MR performance of second and fourth grade boys and girls reported that gender differences favoring boys were only for the fourth-grade students, but not for the second-grade students. In addition, fourth-grade boys performed better than second grade boys in all stimulus conditions, whereas fourth-grade girls outperformed second-grade girls only in familiar figures. Ruthsatz et al. (18) reported that gender differences among fourth-grade students, in favor of males appeared only at the difficult items but not at the easy items. It seems that across their life span, boys' improvement rate in MR is faster than girls' improvement rate, especially for difficult figures. 

*Gender and strategy preference in MR.* A relation between gender and global/local tendency was found for first grade children who completed two-dimensional MR test and a Global-Local Judgment Task (8); boys were more accurate and showed a more global tendency than girls (3). Gender differences in strategy were also found among 10- to 11-year-old boys and girls. For MR task, girls used less efficient strategies than did boys such as analytic strategies or guessing (23). In addition, for secondary school students (24) and for adults (6), the interaction between gender and strategy showed that males tend to use the global strategy, whereas females tend to use the local strategy. 

Nevertheless, among adults, Nazareth et al. (7) found no significant gender differences in strategy selection. Instead, the flexibility to switch between strategies according to the target requirements was found to improve performance, and males had higher strategy flexibility compared with females (7). In addition, gender differences in favor of males were found in MR trials that could be solved by orientation independent strategies as well as by MR strategy. However, when participants were guided to use the orientation independent strategy, gender differences were no longer evident. Therefore, it is possible that gender differences in MR tasks accumulate not only because of global or local processes, but also because of discovering and applying of alternative solution strategies (13).

*Strategy preference and WM in MR.* WM is related to information processing, managing attention to relevant information and problem-solving, from infancy to old age (25, 26). WM capacity was found to improve from childhood to adolescence and therefore, may explain the increased connection between gender and strategy with increase of age (27).

WM [XMLmind] has a significant influence on the accuracy in MR tests among children (28) and adults (29). In addition, among adults WM was found to mediate the relationship between gender and spatial ability across two-dimension MR tests (29).

Spatial WM represents a crucial cognitive process that is essential for goal-directed action, decision-making and learning performance (26). In a recent meta-analysis Voyer, Voyer, and Saint-Aubin (30) reported that spatial WM explained male advantage over female in spatial tasks and that this advantage increased between childhood and adulthood. In our research we examined the spatial WM capacity of children, assuming it may mediate, at least partially, the interaction between gender, strategy and MR performance. 

### Eye movements, gender and cognitive strategy

Eye movements' data reflect attention shifting and provides an indication of the cognitive processes, beyond behavioral parameters of reaction time and accuracy (31, 32). The main parameters that are being used for eye-tracking studies among adults (7, 26, 33) and children (34) are fixations and saccades. Fixations are pauses over informative areas of interest (AOI). AOI are areas that include the target stimuli and/or the answers. In general, researchers' interest is in observing the eye movement pattern in AOI, over areas that do not include any stimulus. The fixations in the AOI are measured by number, location and duration of the fixations (26). A saccade is a quick, simultaneous movement of both eyes between phases of fixation, representing transitions (shifting) of gazes from one region to another. Saccades are measured by duration, latency and number of transitions within or between AOI (26). Dwell time is the sum of duration from all fixations that dwelled on the AOI. 

Higher fixation duration and frequency indicate on a higher cognitive effort (35). Longer fixations on correct answer compared with distractors, reflected greater distinction between right and wrong alternatives (36). Longer response time and smaller saccade amplitude were found for MR mirrored stimuli, compared with identical stimuli, reflecting higher cognitive demand (37). Nazareth et al. (7) found two distinct eye-movement patterns during MR: a fixation of eye-pattern (i.e., low within and between object eye movements) and a switching of eye pattern (i.e., high within and between object eye movements). The switching eye pattern, indicative of a local strategy, was related to higher MR performance. The fixation eye-pattern was indicative of a global strategy; the amount of information to be processed in a global approach is higher and therefore the fixation time is longer (7, 38). Fitzhugh, Shipley, Newcombe, McKenna, and Dumay (39), on the other hand, found that participants with low MR ability showed a higher number of fixations, a finding which was attributed to their local scanning. In contrast, participants with high MR ability had lower number of fixations, a finding which was attributed to a global scanning. 

Men [XMLmind] women allocated visual attention in a similar way, spending more time in MR task observing the target than the possible answers. When men were more accurate than women on the MR test, their advantage was reflected in more useful scanning pattern, as they spent more time observing the correct answers and had shorter fixations on distractors, compared to women (36). On the other hand, Scheer et al. (33), used a MR test that did not reveal gender differences in accuracy or reaction time. Similarly, no gender differences were found in the MR processes, as both men and women showed similar number of fixations and saccades' latency. Nevertheless, gender differences were found for the visual search (at an angular disparity of 0°), as men use a holistic strategy and women a piecemeal strategy (33). Nazareth et al. (7) found gender differences in accuracy in favor of men, yet men and women did not differ in their scanning strategy, as they both used a local strategy. Nevertheless, men demonstrated higher strategy flexibility than women, a finding which may explain men’s advantage in the accuracy measure. 

In our study, we used two main parameters: dwell time and transitions. We analyzed the total dwell time of all AOI (target stimuli, three distractors and one correct answer) and the dwell time of the correct answer versus all answers. Longer total dwell time reflects longer fixation time and/or higher number of fixations resulting in higher dwell time. Because dwell time and transitions represent a longer and more detailed observation (26, 35, 36, 39), we assumed that lower total dwell time represents more global scanning pattern. On the other hand, higher dwell time on the correct answer compared with dwell time on distractors indicates on more global scanning pattern, as the amount of information to be processed on the correct versus incorrect answers is higher (36, 38). We analyzed also the number of transitions (i.e., the number of times the eyes’ focus moved from one AOI to another, within each problem). A larger amount of transitions reflects a more specific and detailed observation (7, 27, 38, 40) and therefore we assumed that higher number of transitions represents more local scanning pattern. 

The focus of the current study was on the relation between gender, cognitive strategy and task difficulty level and between children's accuracy, reaction time and eye movement measures in a MR task. The use of eye movements variable, (an objective physiological measure) allows expanding our knowledge of the behavioral parameters (i.e., accuracy and reaction time). Our sample was composed of fourth grade boys and girls who completed a computerized version of the MR Windows test (CWMR, 39) while their eye movements were monitored. 

### 1.6 Hypotheses

Due to the contradicting finding regarding gender differences and cognitive strategy preference in different MR tasks, in the current study we focused on children’s performance (15), using a two-dimension MR test (3). In the following we present our hypotheses. The hypotheses regarding the combined strategy are based on the research of Nazareth et al. (7), showing that the flexibility to switch between strategies (e.g. to use both local and global strategies) improved performance and that men had higher strategy flexibility than women (7).

*Hypotheses regarding the behavioral measures in the CWMR test:*

1. Boys will achieve higher accuracy scores on the CWMR test than girls. The advantage of boys over girls will be more articulated in difficult compared to easy MR tasks. This hypothesis is based on earlier findings of Tzuriel and Egozi (3, 14), Neuburger et al. (15) and Ruthsatz et al. (18).

2. Participants who use a global strategy or a combined strategy will show higher accuracy than participants who use a local strategy. As regard to reaction time, participants who use a global strategy will show shorter reaction time than participants using a local or combined strategy. This hypothesis is based on the finding of Khooshabeh et al. (5) showing that good imagers rotate complete figures faster, more accurate and in a holistic manner, compared with poor imagers, who were found to rotate these objects in a piecemeal way. 

3. There will be a significant gender by strategy interaction for processing MR tasks; boys will report on using more a global or combined than a local strategy, whereas girls will report on using more a local than a global or combined strategy. This hypothesis is bases on an earlier study showing that boys tend to use a global strategy as compared with girls who tend to use a local strategy (3, 24). It is also based on findings of Nazareth et al. (7) who reported that boys have more flexibility switching between strategies than girls.

4. Spatial WM will be positively correlated with MR accuracy.

*Hypotheses regarding eye movement measures:*

5. An efficient eye movement pattern will be reflected in low total dwell time (35), high dwell time on correct versus incorrect answers (36), and low number of transitions within and between AOI (37). An efficient eye movement pattern will be found for:

a. The easy compared with the difficult level of MR tasks.

b. Boys compared with girls. This hypothesis is based on the finding of Fitzhugh et al. (37) regarding eye movements (see also hypotheses 2 and 3 above). 

c. Children reporting use of a global strategy compared with children reporting a local strategy. Children with a combined strategy will show in general higher efficient eye movement pattern than the local group but lower than the global group. 

*Hypothesis regarding prediction of MR accuracy and reaction time by eye movement measures:*

6. Eye movement measures will predict MR accuracy and reaction time beyond variables of gender, strategy and WM. Higher MR accuracy will be predicted by lower total dwell time, less transitions on AOI, and higher dwell time on the correct answer. Higher reaction time on MR tasks will be predicted by higher total dwell time and more transitions on AOI. 

## Methods

### Participants

The sample of the current research comprised 83 fourth grade children (44 boys and 39 girls), recruited from Israeli public schools. Children's age ranged from 9:03 years and months to 10:03 years and months (M = 9.81 years, SD = .29), with no significant age difference between boys and girls, F(1, 80) = 1.08, p = .08. All the children who participated in this study had no history of developmental problems, could maintain visual attention that was measured by calibration and validation procedure, and were familiar with computer programs. Most participants were right-handed. Yet, left-handed participants were not excluded from the test since the answers were given orally and did not require hand movements. The study was approved by the Ethics Committee of the university and a signed consent was received by the children's parents.

### Materials

**Figure 1.**
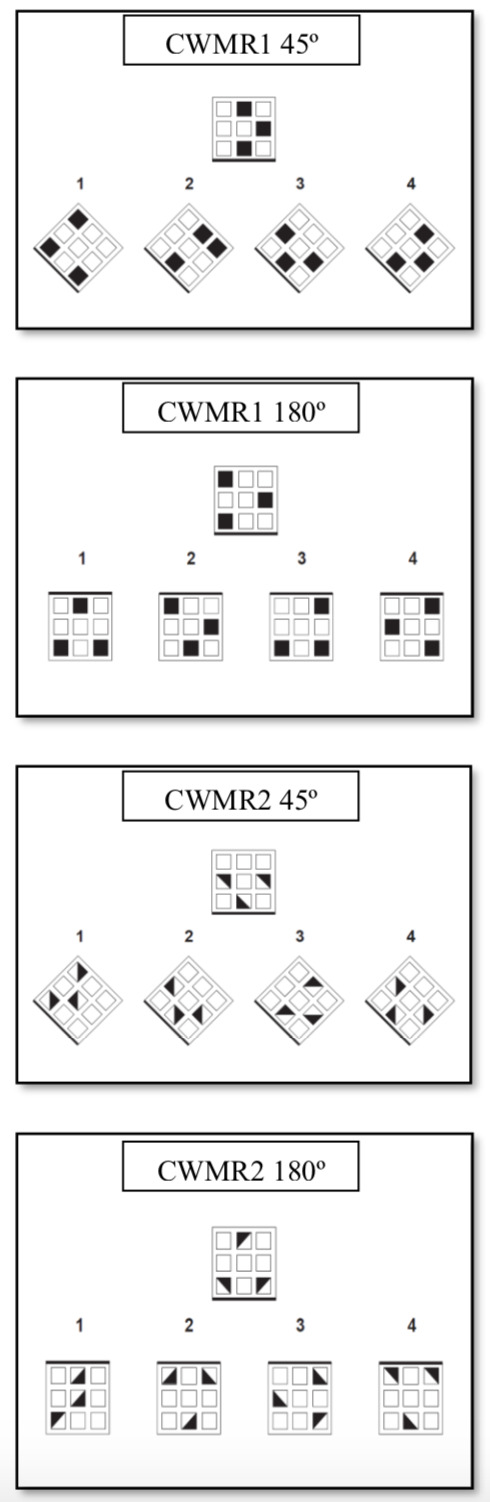
Mental Rotation Items from the CWMR (The heavy black line in each square is in red color in the test. The correct answers are 4, 4, 4, and 2, respectively).

2.2.1 The Computerized Windows MR Test (CWMR). The CWMR test (41) is based on the Windows Test (3) and the Mental Rotation subtest of the Cognitive Modifiability Battery (CMB, 14, 19, 42). It contains 24 computerized slides with MR problems that are presented to the child one at a time. At the top of each slide – target area – there is a model figure of a ‘‘square with windows’’ arranged in a 3 x 3 pattern (9 windows); three windows are “closed” (blackened) whereas six are “open”. At the bottom of each slide – answers area – there are four turned-about “houses”, one is the correct answer and the others are distractors. The child is asked to name the number of the correct answer as fast and accurate as he can, with no time limitation. CWMR test was designed for children in fourth grade and includes two difficulty levels (CWMR1 and CWMR2). The easier level includes three windows that are blackened (square shape), and the more difficult level includes three windows that are half blackened (diagonal shape) and their direction may change inside the square. The items in the difficult level require rotation not only of the window, but also the position of the diagonal half-blackened square within the window. This requirement demands higher level of WM and rotation of two elements rather than one element as in the easy level. For each difficulty level there are two rotation degrees (45º and 180º). Examples for the different difficulty levels and rotation degrees are presented in Figure 1. An explanation slide is presented and read to the child for each difficulty level and rotation degree, followed by a practice example. 

The model “house” is presented in an upright position and the four alternatives are rotated. The clue for the direction of the rotation is given by a red base line drawn at each square (at Figure 1 the red line is presented as a bold line). For each level, there are 12 problems (6 for each rotation degree). Each correctly solved window is given a score of 1 (maximal score = 24). Cronbach alpha reliability was .77. Construct validity was based on the findings of the Windows Test (3) which showed a significant performance decrease as a function of degree of rotation, *F*(2, 111) = 416.80, *p* < .001, *η*_*p*_² = .88, and test level, *F*(1, 112) = 366.70, *p* < .001, *η*_*p*_² = .77.

2.2.2 The Spatial Span subtest (43). In order to measure the correlation between spatial WM and MR ability, children performed the Spatial Span subtest. The Spatial Span subtest of the Wechsler Memory Scale – Revised is an indicator of the visuospatial component of WM. In the test, a board with ten blocks on it is presented to the participants. The experimenter taps the blocks in a specific order and creates a series of spatial patterns, and the participant needs to repeat the same order in the Spatial Span Forward test and the opposite order in the Spatial Span Backward test. Both tests begin with a series of 2 blocks and there are two trials for each series length. The number of blocks increases by one block every two trials. There are 16 trials forward and 16 trials backward, and each correct answer is scored with one point (the score range is 0 to 32). Cronbach alpha reliability coefficient is .86. 

2.2.3 Child Self-Report of Strategy. A simple self-report of strategy was constructed for this study for the MR task, in order to examine the interactions between strategy and MR performance and strategy and gender. After performing the CWMR participants were asked to explain their thinking processes while solving the CWMR problems. Their answers were sorted into three categories: global, local and combined strategy. Previous studies used Self-Report of Strategy as an objective tool for strategy measures in spatial performance (44, 45).

**Figure 2.**
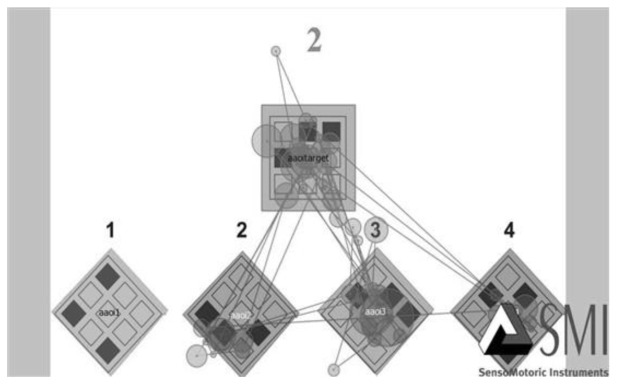
Eye Movement Measures on Slide 2 of the CWMR Test. Aaoitarget = the Target area; Aaoi1 to Aaoi4 = the Answers area; Each line represents transition (saccade), and each circle represents fixation.

2.2.4 Eye movements. The CWMR was programmed in the SMI RED-M remote eye-tracker (SensoMotoric Instruments, Teltow, Germany). Sample rate was 120 Hz with high accuracy of 0.8°. Eye movements were recorded by a camera with an ultra-light source that was placed in front of the laptop screen (Size 41 X 23.1 cm), below eye level. Participants were seated approximately 60 cm in front of the screen. An additional computer was used by the experimenter to monitor the stimuli and the eyes path during the experiment. Prior to the eye tracking tests, all participants went through a 5-point calibration procedure which provided a spatial resolution of 0.1°. During calibration, the participants were asked to look at the center of a dot that was moving on the screen and stopping on 9 locations (all locations were previously set; the first 5 locations were used for calibration and the subsequent locations for validation). If the accuracy was beyond the threshold of 0.8°, calibration and validation were repeated. For nine children this procedure could not have been conducted successfully, due to problems related to eye impairments or attentional deficits. Therefore, these nine subjects were excluded from the study. Participants viewed 24 question items of the CWMR test, each for as long as it took to generate an answer, and answered orally, by naming aloud the number of the answer they deemed correct. The answer was written by the experimenter. Oral answers were preferred over written answers to prevent children from moving their eye focus away from the screen. The eye movement parameters that were measured and analyzed in this experiment were: dwell time and number of transitions. Total dwell time - the sum of durations from all fixations that dwelled on the AOI. In our study, we analyzed the dwell time at 5 AOI: target stimuli, three distractors and one correct answer, and of the correct answer versus all answers. Higher number of fixations and longer fixation duration result in higher dwell time, representing a longer and more detailed observation. Transitions - the number of times the eyes focus moved from one AOI to another. In our study we analyzed the number of transitions within each slide. A larger amount of transitions reflects a more specific and detailed observation. The eye movement measures of the CWMR test can be seen in Figure 2. Aaoitarget- represents the target area, Aaoi1 to Aaoi4 represent the Answers area. Each line represents transition (saccade), and each circle represents a fixation. 

### Procedure

Before conducting this research, a pilot study was carried out in order to corroborate the level of the test with the children's age and performance, and to examine the participants' ability to understand a computerized version. In the current research, the study's procedure was explained to all participants prior to beginning the study. The children were told that they will be tested on a spatial task while monitoring their eye movements, but they were not told that gender differences will be measured. In order to ensure that all participants were familiar with computer programs, they were asked how many hours a week they play on the computer. All participants play on the computer during the week. All children were tested individually in a quiet schoolroom. First, they were given an instruction and practice stage on the CWMR test, followed by the CWMR test, the Self-Report of Strategy and the Spatial Span subtest, in that order. The instructions for the CWMR test were presented on a printed document containing standard instructions. The instructions were read by the experimenter and the children practiced two sample items for each of the difficulty levels. During the explanation and the practice items, children were permitted to request clarification if they did not understand the questions or the instructions. The participants were then seated on a chair in front of the computer, approximately 60 cm from the eye tracker system, and conducted a collaboration and validation procedure. Participants who succeed in this procedure were administered the CWMR test while their eye movements were monitored. The participants were requested to answer as fast and accurate as possible, with no time limit, and to vocally state the number of the answer they chose. Before each cluster of questions (for each difficulty level and for each rotation degree) a slide was presented showing the difficulty level and rotation degree. These slides were not included in the time and eye movements' calculation. After performing the CWMR participants were asked to explain their thinking processes while solving the CWMR problems. The question asked was: "Please explain to me how you managed to answer these questions. What was your thinking process when answering?" The answers were written by the experimenter, then participants performed the Spatial Span subtest and received a small prize. The children's self-report of strategy were sorted into three categories: global (i.e., rotating the entire shape unit), local strategy (i.e., observing each window separately), and combined strategy (i.e., using both global and local strategies or using the relationship between two windows in order to find the correct answer). The participants' explanations were given to two examiners, in order to examine inter-judge reliability. The findings revealed high agreement (97%) between the judges based on categorical sorting. 

## Results

### MR ability: Behavioral measures

To test the hypotheses of behavioral variables for the CWMR test, the variables of accuracy and reaction-time were each analyzed by mixed ANOVA of 2 x 3 x 2: Gender (2) by Self-Report of Strategy (sorted into three categories: global, local and combined) by Difficulty Level (2). The first two variables are between-subject factors and the last variable is a within-subject factor.

**Figure 3.**
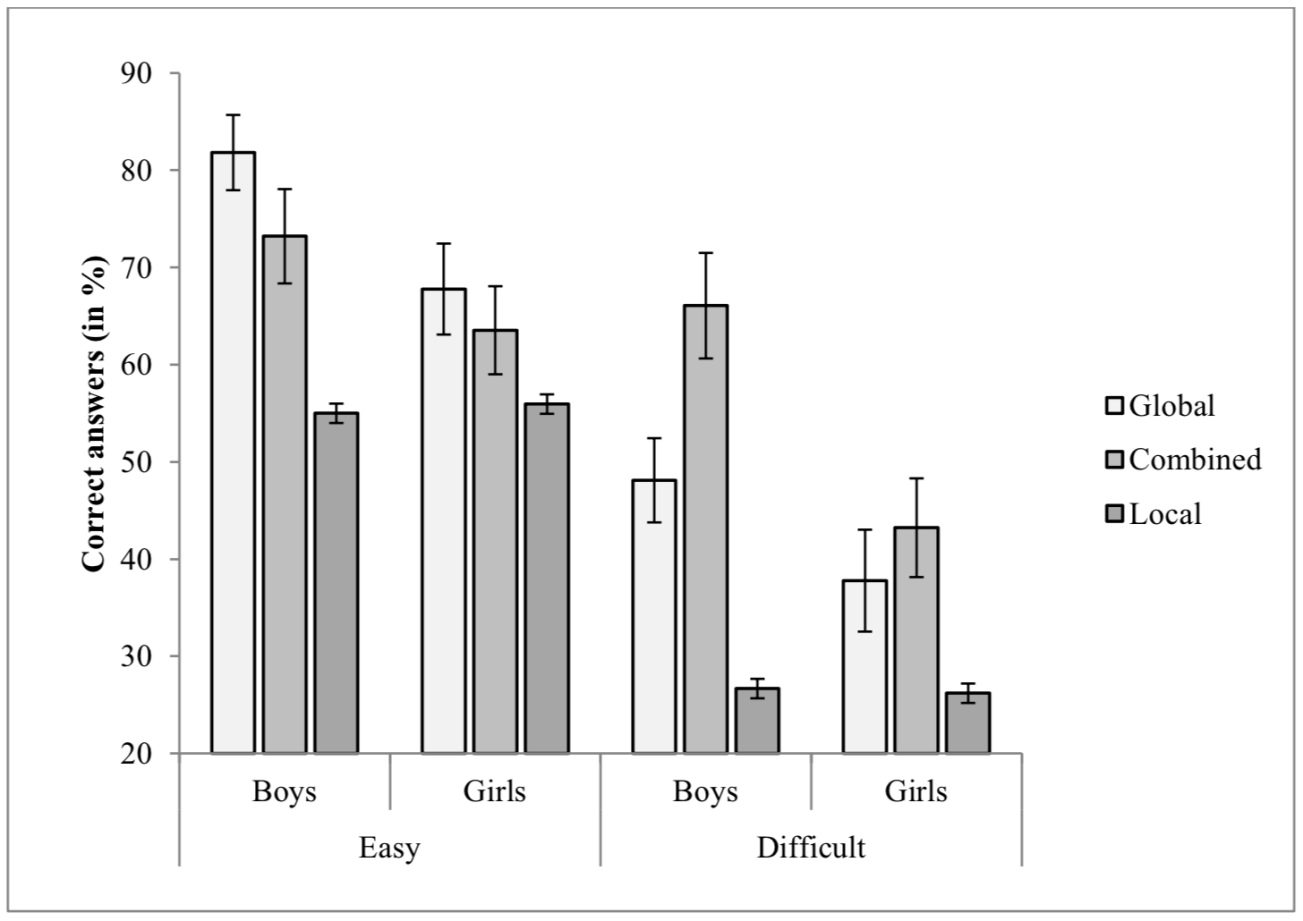
Percent of Correct Answers on the CWMR Test.

3.1.1 Correcty answers. The data was analyzed by mixed ANOVA of Gender X Self-Report of Strategy X Difficulty Level (2 X 3 X 2) with percent of correct answers as the dependent variable. The means and standard errors of correct answers for each subgroup of gender by strategy by difficulty level are portrayed in Figure 3.

The analysis revealed that participants were more accurate at the easy level than at the difficult level, *F*(1, 73) = 92.24, *p* < .001, *η*_*p*_² = .55. Because of multiple testing, Bonferroni correction was chosen, and the significance level was *α* = 0.001. Significant Gender main effect was found, *F*(1, 73) = 5.02, *p* < .05, *η*_*p*_² = .06, indicating that the percent of correct answers was higher for boys (M = 62.97, *SD* = 17.68) than for girls (M = 51.18, *SD* = 17.36). Because of multiple testing, Bonferroni correction was chosen, and the significance level was *α* = 0.028. The effect size of Gender by Difficulty Level was larger at the difficult than at the easy level (see Table 1). The main effect of Self-Report of Strategy was significant, *F*(2, 73) = 6.95, *p* < .01, *η*_*p*_² = .16. Because of multiple testing, Bonferroni correction was chosen, and the significance level was α = 0.001. Post hoc analysis using Bonferroni revealed that the overall percent of correct answers was significantly lower (*p* < .01) for the local group (M = 40.97, *SD* = 11.49), than for the global (M = 60.02, *SD* = 17.11) and combined (M = 60.97, *SD* = 19.40) groups.

No significant interactions were found for Difficulty Level by Gender, *F*(1, 73) = 1.10, *p* =.48, *η*_*p*_² = .01, or Gender by Self-Report of Strategy, *F*(2, 73) = 1.06, *p* =.38, *η*_*p*_² = .03. The interaction of Difficulty Level by Self-Report of Strategy was significant, *F*(2, 73) = 6.87, *p* < .01, *η*_*p*_² = .16. Post hoc analysis using one-way ANOVA for each difficulty level revealed that at the easy level, the overall percent of correct answers was significantly lower (*p* < .01) in the local group as compared with the global group. At the difficult level, the overall percent of correct answers was significantly lower in the local group compared to the global and combined groups (*p* < .05). It should be noted that on the difficult level the effect of Self-Report of Strategy was reduced as compared with the easy level.

**Table 1:**
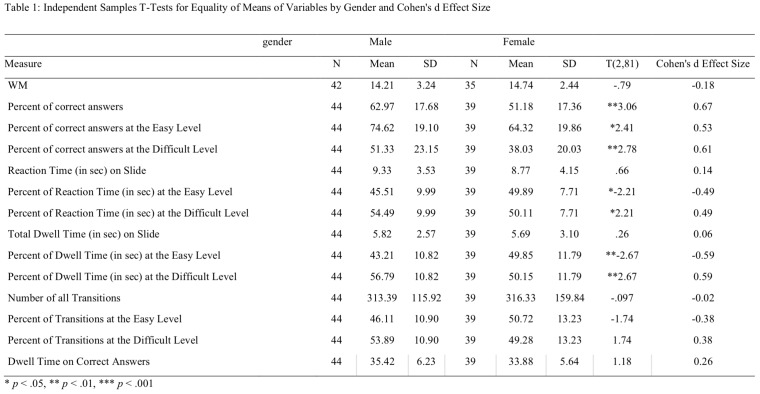
Independent Samples T-Tests for Equality of Means of Variables by Gender and Cohen's d Effect Size

*p < .05, **p < .01, ***p < .001

3.1.2 Reaction time. In order to analyze the reaction time of participants, mixed ANOVA of Gender X Self-Report of Strategy X Difficulty Level (2 X 3 X 2) was conducted. Significant main effect was found for Self-Report of Strategy, *F*(2, 73) = 4.61, *p* < .05, *η*_*p*_² = .11. Post hoc analysis using Bonferroni revealed that the average reaction time was significantly lower (*p* < .05) for the global group (M = 7.96, *SD* = 2.85) than for the combined group (M = 10.44, *SD* = 3.94), but these *groups* did not significantly differ from the local group (M = 9.35, *SD* = 5.51).

No significant main effect was found for Gender, *F*(1, 73) =.01, *p* = .89, *η*_*p*_² = .01. Yet, as can be seen in Table 1, proportionally boy's reaction time was longer on the easy than on the difficult level whereas girl's reaction time was longer on the easy than on the difficult level.

**Figure 4.**
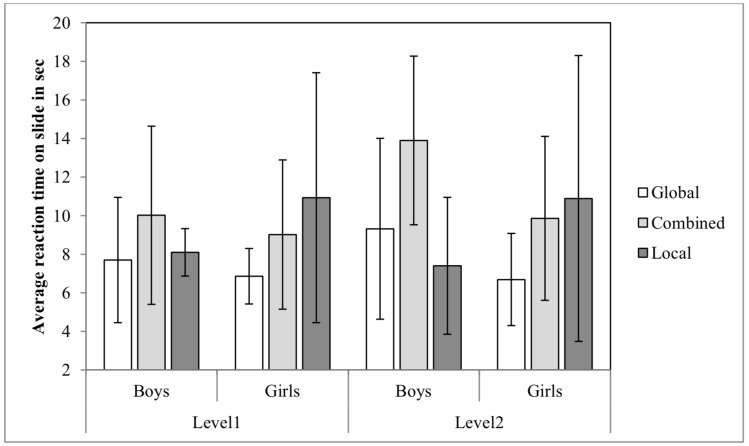
Average Reaction Time on Slide on the CWMR Test.

The interaction between Self-Report of Strategy and Gender was significant, *F*(2, 73) = 3.15, *p* < .05, *η*_*p*_² = .08. The interaction indicates that boys who reported on using the local strategy had shorter reaction time than boys who reported on using the combined strategy, whereas girls who reported on using the global strategy had shorter reaction time than girls who reported on using the local strategy (see Figure 4).

No significant differences were found for Gender by Self-Report of Strategy, *χ* ^2^ (2, N = 79) = 1.70, *p* = .43. 

In summary, the first hypothesis was supported: gender differences were found for the accuracy measure. Boys were more accurate than girls at both difficulty levels, and the gender effect size was larger for the difficult than for the easy level of MR. In accordance with the second hypothesis, strategy was found to effect accuracy and reaction time; children who reported on using the local strategy were less accurate than children who reported on using the global or the combined strategy. Nevertheless, regarding reaction time, only part of the second hypothesis was supported. Children who reported on using the combined strategy had longer reaction time compared with children who reported on using the global strategy; no significant difference was found between the local and the global groups. The interaction between gender and self-report of strategy (third hypotheses) was insignificant.

3.1.3 Pearson correlations between accuracy and spatial WM. In order to analyze the fourth hypothesis, Pearson correlations were carried out between accuracy and spatial WM for each gender separately, for each strategy group separately and for all children together. The results (see Table 2) indicate a positive correlation between spatial WM and accuracy at the easy difficulty level of the CWMR test. This correlation was significant for the total sample and for girls. Significant correlation was also found for children who reported on using the local strategy. The relation between gender and WM was measured by t test. The WM capacity did not differ by gender, *t*(75) = -.79, *p* = .43.

**Table 2:** Pearson Correlation between Accuracy in the CWMR Test and Spatial WM in the Total Sample, in Each Group of Self-Report of Strategy, and for Boys and Girls.

Group	Total Score	Easy Level	Difficult Level
Total Sample	.14	.25*	.02
Local	.29	.60*	-.12
Combined	.18	.22	.09
Global	.23	.13	-.07
Boys	.17	.23	.06
Girls	.20	.35*	.06

*p < .05, **p < .01, ***p < .001

### Mental rotation ability and eye movement measures

Two primary dependent measures reflecting eye movements were analyzed: (a) overall time on AOI (in seconds) and (b) the number of transitions from one region to another. Each slide consists of 5 AOI: the top area containing the target stimuli (problem) and the bottom area containing four sub-regions: three distractors and one correct answer. Like the behavioral measures, the eye movement measures were analyzed by mixed ANOVA of 2 x 3 x 2: Gender (2) by Self-Report of Strategy (3) by Difficulty Level (2). The main effects and interactions between Gender, Difficulty Level and Self-Report of Strategy, for all eye movement measures, are summarized in Table 3.

**Table 3:**
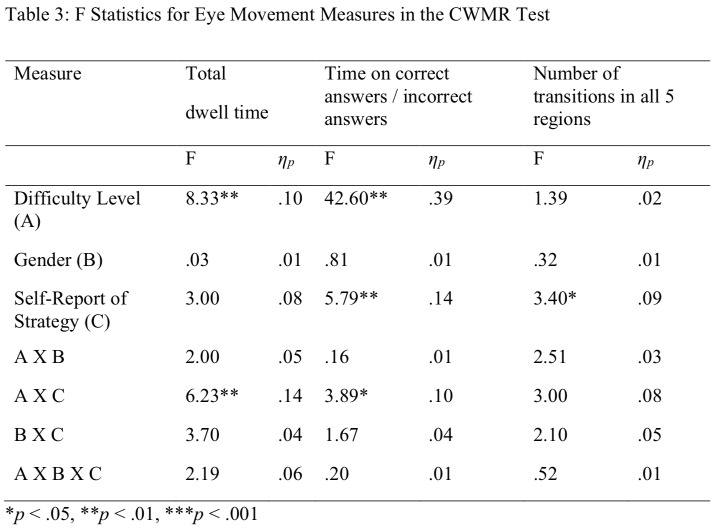
F Statistics for Eye Movement Measures in the CWMR Test

*p < .05, **p < .01, ***p < .001

**Figure 5.**
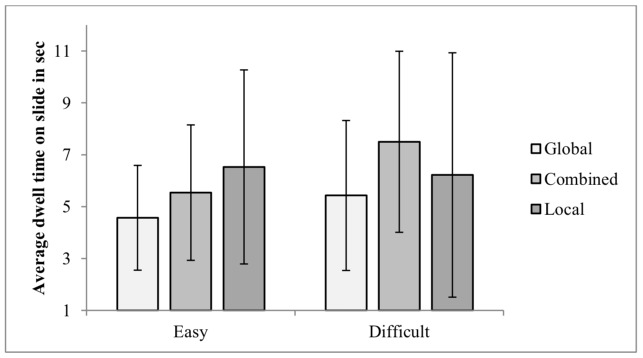
Average Dwell Time on Slide on the CWMR Test.

3.2.1 Total dwell time. Participants total dwell time was significantly lower at the easy level (M = 5.23, *SD* = 2.63) compared with the difficult level (M = 6.34, *SD* = 3.53). Because of multiple testing, Bonferroni correction was chosen, and the significance standard was α = 0.005. The interaction between Self-Report of Strategy by Difficulty Level indicated that the local group had significantly higher dwell time at the easy level than at the difficult level, whereas the global and combined groups had longer dwell time at the difficult level compared with the easy level (see Figure 5). As can be seen in Table 1, boy's total dwell time was proportionally longer for the difficult than for the easy level, whereas girls' total dwell time was proportionally longer for the easy than for the difficult level. 

3.2.2 Proportional time on the right answer/time on all other options. Participants had significantly longer dwell time on the correct answer at the easy level (M = 39.88, *SD* = 8.53), compared with the difficult level (M = 29.87, *SD* = 9.32). Post hoc analysis for the significant difference in Self-Report of Strategy group using Bonferroni indicated that the overall percent of dwell time on the correct answers of the local group (M = 30.01, *SD* = 5.09) was significantly (*α* = 0.005) lower than for the combined group (M = 36.27, *SD* = 6.55), but not from the global group (M = 34.97, *SD* = 5.33). 

**Figure 6.**
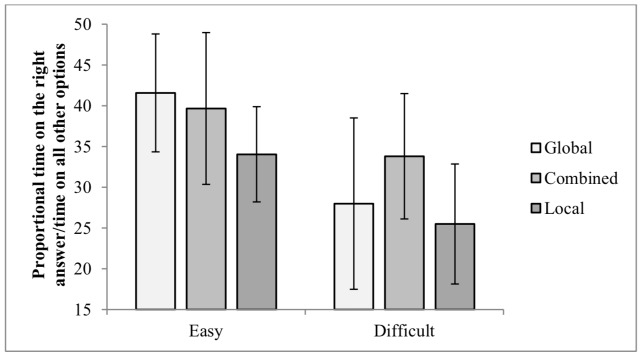
Proportional Time on the Right Answer/Time on all other Options on the CWMR Test.

Post hoc analysis of the significant interaction between Self-Report of Strategy and Difficulty Level revealed that at the easy level, the global group spent more time on the correct answer than the local group. At the difficult level, the combined group spent more time on the correct answers than local and global groups (see Figure 6).

**Figure 7.**
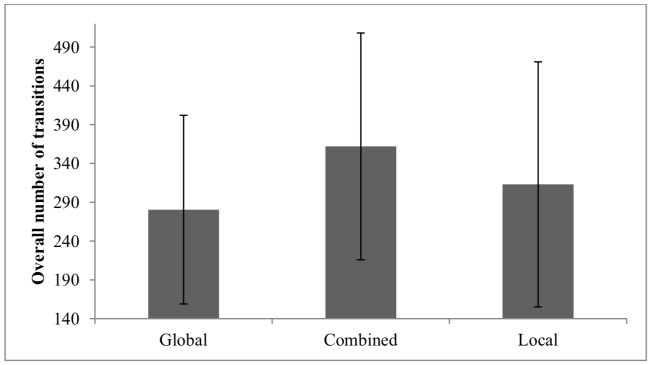
Overall Number of Switches on the CWMR Test.

3.2.3 Overall number of transitions across all five regions of interest. The main effect of Self-Report of Strategy was significant. Follow-up analysis using Bonferroni revealed that the overall number of transitions was significantly lower (*p* < .05) for the global group than for the combined group (see Figure 7). The effects size of gender for all behavior and eye movement parameters are presented in Table 3.

In summary, regarding the fifth hypothesis, an efficient eye movement pattern was found for the difficulty level (part a); higher difficulty level increased the total dwell time and decreased the dwell time on the correct answer, but no significant difference was found for the number of transitions. In contrast to the fifth hypothesis (part b), boys and girls did not differ in their eye movement pattern. For the Self-Report of Strategy (part c), children with a global strategy showed a more efficient eye movement pattern than children with a local strategy. As for children reporting a combined strategy, they showed in general higher efficient eye movements than children in the local group but lower than children in the global group. 

Children who reported on using the global strategy had less transitions compared with children who reported on using the combined strategy. Children who reported on using a combined strategy spent more time on the correct answers (compared with the distractors) than children who reported on using the local strategy. The interaction between Self-Report of Strategy and Difficulty Level revealed that the local group had longer total dwell time on the easy level, whereas the global and combined groups had longer total dwell time on the difficult level. Moreover, the local group had shorter dwell time on the correct answers for both difficulty levels compared with the global group at the easy level and with the combined group at the difficult level (see Figure 6).

### Prediction of MR accuracy and reaction time by eye movement measures

To examine the prediction of MR accuracy and reaction time by eye movement measures (hypothesis 6), we carried out two hierarchical regression analyses of accuracy and reaction time as criterion variables. In step 1 we introduced the variables of Gender, Self-Report of Strategy, and WM as controlled variables. In the second step we introduced the eye movement measures (i.e., total dwell time, number of transitions and proportional time on the correct answers) as predictive variables. The findings are presented in Table 4.

**Table 4:**
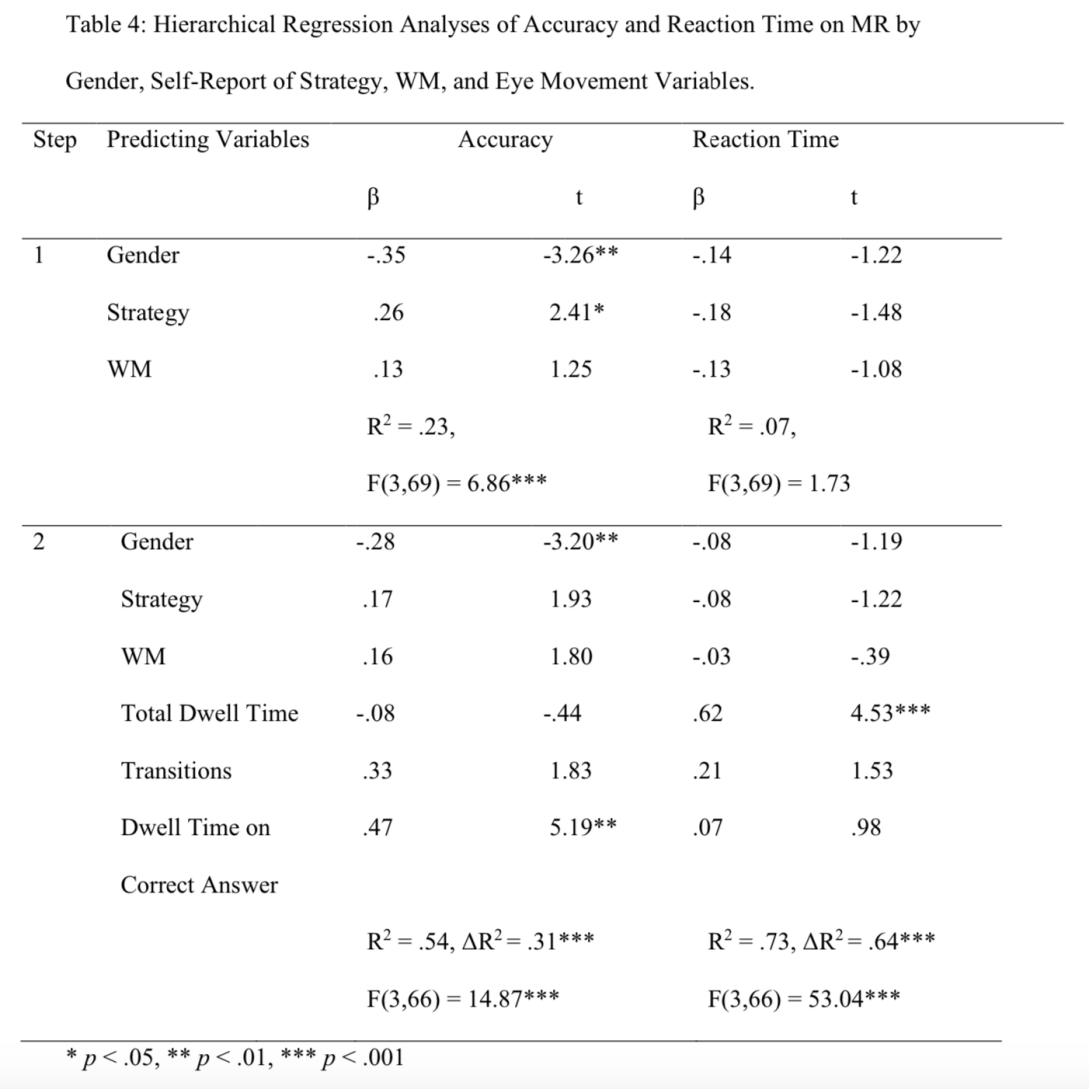
Hierarchical Regression Analyses of Accuracy and Reaction Time on MR by Gender, Self-Report of Strategy, WM, and Eye Movement Variables.

*p < .05, **p < .01, ***p < .001

Prediction of Accuracy. The findings shown in Table 4 indicate that from the three eye movements variables dwell time on the correct answer significantly and positively predicted accuracy. The contribution of eye movements indicators was 31% above and beyond the variables of Gender, Self-Report of Strategy and WM. It is interesting to note that Gender predicted accuracy both, before and after introducing the eye movement measures; boys scored higher than girls. Self-Report of Strategy predicted positively accuracy only in step 1 of the analysis, before introducing the eye movement measures; children with global strategy scored higher than children with local strategy. 

Prediction of Reaction Time. The Total Dwell Time was the only significant variable predicting positively reaction time. The findings (Table 4) indicate that from the three eye movements variables Dwell Time on the Correct Answer significantly and positively predicted reaction time. The contribution of eye movements was 64% above and beyond the variables of Gender, Self-Report of Strategy and WM; none of the controlled variables predicted significantly reaction time. 

## Discussion

MR is known to be affected by various variables, such as task demands, gender, strategy and age (3, 15, 24). In the current study we focus on the effect or association of each of these factors and the correlation between them regarding MR performance among children. Moreover, we examined the prediction of accuracy and reaction time in MR tasks by eye movements variables. The focus however is on eye movement measures, which reflect thinking processes and enable both intensive and extensive understanding of the effects of strategy, gender and difficulty level of MR task. 

### Gender differences

In accordance with the first hypothesis and with the findings of previous studies (6, 20), in the current study boys achieved significantly higher accuracy scores on the CWMR test than girls. The advantage of boys over girls was found for both CWMR difficulty levels. Also, as expected, the gender effect size was higher at the difficult compared with the easy level. These finding support previous findings, showing that gender differences in MR exist already in childhood (e.g., 3, 14, 22), and increases with difficulty level (18).

Boys and girls did not differ in their reaction time or total dwell time, yet boys spent proportionally more time observing the difficult than the easy items, whereas girls spent proportionally more time observing the easy than the difficult items. A possible explanation for these findings is that for boys the problems of the easy level did not require a detailed observation, whereas for girls these items were experienced as more difficult, and therefore required more observation time. On the difficult level, it is possible that for boys the problems were experienced as more difficult (than the easy problems), and therefore required proportionally more observation time than for the easy problems. In contrast, for girls the problems on the difficult level were too hard, and therefore they did not used detailed scanning, but rather used a guessing approach.

In the fifth hypothesis (part b), we assumed that different cognitive strategies will be used by boys and girls and that it will be reflected in different eye movement patterns. In contrast to this hypothesis, the findings revealed that boys and girls did not differ in their scanning pattern. It seems that the behavioral gender differences in accuracy do not stem from the use of different scanning strategies. Similar findings were reported for adults by Nazareth et al., (7) and by Scheer et al., (33). In their studies men and women did not differ in their eye movement pattern.

### Global/Local strategy

After performing the CWMR test, participants explained their thoughts and thinking processes while solving the test. The answers were classified into three strategy categories: Global, Local, and Combined strategy. Our findings indicate that the strategy participants reported using was associated significantly with their accuracy, reaction time and eye movement pattern. 

As expected in the second hypothesis and like previous studies (5, 6, 7, 14), participants who used global and combined strategies showed higher accuracy than participants who used a local strategy. Participants who used a global strategy had faster reaction time than participants who used the combined strategy. Nevertheless, in contrast to the second hypothesis and due to the strict significance level of Bonferroni, the difference between the reaction time of the global and the local groups was not significant. The findings seem to be reasonable in that the combined strategy, which includes both local and global strategies, requires more time than the global strategy alone. 

 The findings on eye movement of children with different strategies (fifth hypothesis, part c) supports partially the fifth hypothesis (part c). As expected, the global group had an advantage over the local group; the global group had longer dwell time on the correct answers at the easy level than the local group. Yet, in contrast to our hypothesis and to previous findings (39), no significant differences were found between the local and the global groups on total dwell time and number of transitions measures. In addition, and as expected, children who reported on the combined strategy had an advantage over the local group, having longer dwell time on the correct answers. Children who reported on using the global strategy had an advantage over children with a combined strategy; they showed lower number of transitions. 

Simultaneous [XMLmind] of the behavioral and the eye movements findings revealed that the local strategy was the least effective strategy. The global strategy was found to be more efficient than the local strategy. These findings support previous findings showing that the global strategy is more efficient that the local strategy for MR tasks (14, 39). The combined strategy had a speed-accuracy tradeoff. Children in the combined group were more accurate and had longer dwell time on the correct answers, as compared with the local group, and showed longer reaction time and higher number of transitions than the global group. These findings emphasize the need to consider simultaneously behavioral and eye movements measures. 

The examination of children using a combined strategy is important as there are no known studies referring to eye movements of children using a combined strategy. The only two studies supporting the effectiveness of a combined strategy for MR accuracy indicate that switching strategies in a flexible way improved MR performance (5, 7). Our study shows further that the combined strategy is intimately related to eye movement pattern. It seems that the unlimited time given to solve the MR tasks allowed children in the combined group to scan both global and local elements of the figure. This unique scanning pattern raised simultaneously their accuracy and reaction time. 

Our findings should be taken cautiously as children were asked to explain their thinking processes only at the end of the test. It is possible that participants changed their strategy several times during the testing procedure, and even during the same slide (13). We assume that in general children reported on the main strategy they have used or on using both strategies (combined group) as some of them indeed reported using. However, it is possible that some of the children used more than one strategy and reported only on one. Moreover, the interactions found between the difficulty level and the eye movement measures may indicate on the need to switch between strategies according to the task demands. All these concerns require further research to examine the effects of strategy on accuracy and eye movement pattern.

### Gender and strategy

In contrast to the third hypothesis, no significant Gender by Strategy interaction was found for the accuracy measure. It should be noted that in previous studies, the Gender by Strategy interaction was found to be affected by age (3, 15, 24, 46), and by the task characteristics (3, 47); the higher the age or task difficulty the higher was the interaction effect. The impact of age on Gender by Strategy interaction can be explained partially by WM capacity, which increases with age (27). Spatial WM explains male advantage over female in spatial tasks, such as MR tasks, and the magnitude of this advantage was found to increase with age (30). In the current research, boys and girls did not differ in their spatial WM capacity, a finding which may contribute to the insignificant Gender by Strategy interaction. 

The impact of task characteristics on the Gender by Strategy interaction can be explained by difficulty level of MR tasks. Unlike our findings, Tzuriel and Egozi (3) reported a significant gender by strategy interaction; boys were more accurate and more global than girls. The difference between the findings of the two studies might be attributed to the difficulty level of the MR tests used in both studies. In Tzuriel and Egozi’s (3) study children were presented the easy level of the original Windows Mental Rotation Test (i.e., CWMR1), whereas in the current study the difficult level was included. Thus, it is plausible to assume that in the current study the global strategy was not enough to grasp all the different details of the shape, and therefore there was a need to use a local strategy as well, hence the reporting of use of both strategies (i.e., combined group). 

A significant Gender by Strategy interaction found for the reaction time measure, indicates a different pattern for boys and girls. Boys reporting use of a local strategy, spent less time than boys reporting use of a combined strategy. This finding might be explained by the need of boys in the combined group to make decisions what kind of strategy to use to solve the difficult MR problem. The findings for girls however coincide with previous findings showing that a global strategy produce a shorter reaction time than a local strategy (5). It should be emphasized that this is a post-hoc explanation which raise the question of why there are gender differences regarding the relation between strategy and reaction time, a question that is open for further research. 

For all eye movement measures no significant interaction was found between gender and strategy; a finding which does not support our expectations. The findings of the eye movement measures support the findings related to accuracy, and strengthens the claim that among children, gender does not have a definite effect on the tendency to use one strategy over the other. 

### Difficulty level

The difference in the difficulty level, reflected in higher accuracy at the easy than at the difficult level, enabled a wider understanding of the eye movement patterns that are required when an effort to solve MR tasks arises. In accordance with the fifth hypothesis (part a), and similar to previous findings (26, 35, 36, 39), an increase of the difficulty level increased the total dwell time and decreased the dwell time on the correct answers. However, in contrast to the fifth hypothesis (part a) and to previous studies (7, 37, 38), the number of transitions did not differ between the easy and difficult levels. 

The significant Difficulty Level by Strategy interaction indicates that for the easy level the global strategy is the most effective strategy, whereas for the difficult level the combined strategy is the most effective strategy. It seems that for the easy level the global strategy was enough in order to reach accuracy. Yet, for the difficult level, the global strategy by itself was not enough, and there was a need to add a specific scanning of the shape’s components in order to reach high accuracy. A variable that might explain the benefit of using a combined strategy is cognitive flexibility. Previous findings revealed that flexibility improved accuracy in MR tasks (5), and that men were more flexible and accurate than women (7). In the current research, we assume that flexibility was needed in order to switch from one strategy to another, according to the task demands. Moreover, it is possible that boys were more flexible than girls, and adjust their scanning pattern to the task demands, and therefore showed higher accuracy. 

Another explanation for the use of different strategies in easy and difficult MR tasks is related to WM capacity. Children can use the global strategy only when the number of components that needs to be remembered in a shape is low (e.g., easy MR task). In contrast, when the number of components in a shape is high (e.g., difficult MR task), children rely on a local scanning strategy as well. Support for this explanation might be found in the significant correlation between the MR accuracy and WM capacity (r = .25) only for easy MR tasks but not for difficult MR tasks (r = .02). The easy level requires a moderate amount of global perception, which requires a reachable amount of WM, hence the significant positive correlation between the two variables. This finding indicates that for the easy level, WM capacity is critical for success. In contrast, the difficult level requires an unreachable amount of WM and therefore children need to combine both strategies and take more time in order to perform accurately. 

This insight became possible thanks to the combination of using simultaneously the behavioral, eye movements and WM measures. Although the interaction between WM, difficulty level and strategy require further research, the current findings may explain some contradicting findings regarding MR accuracy and strategy. Consideration of these variables may help in explaining the differences among children and adults using the local versus the global strategy, the differences between children's performance in different MR tasks, and the contradicting finding regarding the correlations of MR and WM as a function of cognitive strategy. In addition, the age in which gender differences in MR emerge may be mediated by the difficulty level of the task; when the difficulty level is too high a floor effect prevents gender differences from being revealed. It should be noted that the CWMR test included two difficulty levels, and two rotation degrees for each difficulty level and that before each task there was a slide that informed the child about the difficulty level and the rotation angle. These slides were needed in order to present the task demands and to ensure that the child knows what he/she needs to do. This procedure might have an influence on the child’s expectations and strategy.

### Eye movement measures

The eye movement findings contributed significantly to our understanding of performance in MR tasks. They reflected the task difficulty level, the differences in the scanning pattern for each strategy and the appropriate strategy for each difficulty level. They also revealed similar thinking patterns between boys and girls on the CWMR test, despite the difference in accuracy. 

In the sixth hypothesis, we expected eye movement parameters to predict accuracy and reaction time. We examined the unique contribution, for accuracy and reaction time separately using three eye movement parameters: total dwell time, dwell time on the correct answer and number of transitions. We applied a hierarchical regression analysis to examine the unique contribution of eye movement measures to accuracy and reaction time beyond the variables of gender, strategy and WM. 

The sixth hypothesis was partially confirmed. As expected, dwell time on the correct answer predicted positively accuracy, and total dwell time predicted positively reaction time. In contrast to the hypothesis, total dwell time did not predict accuracy, and the number of transitions did not predict accuracy and reaction time.

Similar findings were found by Alexander and Son (36), showing that men and women had a similar scanning pattern, though men had longer fixation on the correct answer and were more accurate than women.

Since dwell time on the correct answer was not found to be related to any specific strategy it is plausible to assume that accuracy is determined mainly by the ability to switch between eye movement scanning patterns according to the task demands. Support for this idea was found by Nazareth et al. (7) who showed that the flexibility to switch between strategies according to the task requirements predicted accuracy, and that males had higher flexibility in moving between strategies compared with females. 

In the current study, total dwell time was the only measure that predicted reaction time. It should be noted that the eye movement pattern was recorded without a time limit. Time limit was found previously to increase gender differences in accuracy favoring men (48), affect the scanning pattern and restricts participants’ choice of one strategy over another. On the other hand, unlimited time may enable participants to use more than one strategy at a time, and to change their scanning pattern according to the task demands. Therefore, it is possible that the unlimited time used in the current study reduced the effect of the other variables that were included in the regression analyses on reaction time. In summary, the current study shows that eye movement measures had a significant contribution, explaining the variance in both accuracy and reaction time measures among children.

### Conclusion

 In the current study gender differences in MR tasks were found among fourth grade students in favor of boys and increased in accordance with the difficulty level. These differences do not derive from a different scanning pattern, as boys and girls showed similar eye movement pattern. Strategy was found to affect performance. Participants reporting use of a global or combined strategy were more effective in using MR tasks than participants reporting using a local strategy. The effectiveness of eye movements decreased with increase in difficulty level of MR tasks. Boys and girls changed their scanning pattern as a function of task difficulty. Accuracy in solving MR tasks and reaction time were significantly predicted by eye movement pattern.

## Ethics and Conflict of Interest

The author(s) declare(s) that the contents of the article are in agreement with the ethics described in http://biblio.unibe.ch/portale/elibrary/BOP/jemr/ethics.html and that there is no conflict of interest regarding the publication of this paper. 

## Acknowledgements

This research is based on a Doctoral Dissertation of the first author. The study was funded by a grant from, President’s Stipends Fund of Bar-Ilan University. The authors would like to acknowledge Mordecai Katz, Monique Katz and the Moses S. Schupf Fellowship Program for their generous sponsorship and for their support of the Doctoral degree studies and research. 
